# Effects of a 12-month multi-faceted mentoring intervention on knowledge, quality, and usage of spirometry in primary care: a before-and-after study

**DOI:** 10.1186/s12890-016-0220-6

**Published:** 2016-04-21

**Authors:** Samir Gupta, Dilshad Moosa, Ana MacPherson, Christopher Allen, Itamar E. Tamari

**Affiliations:** Department of Medicine, University of Toronto, Toronto, Canada; Division of Respirology, Department of Medicine, St. Michael’s Hospital, Toronto, Canada; Li Ka Shing Knowledge Institute of St. Michael’s Hospital, Toronto, Canada; Ontario Lung Association, Toronto, Canada; Southlake Regional Health Centre, Newmarket, Canada; Department of Medicine and Firestone Institute for Respiratory Health, McMaster University, Hamilton, Canada; Stonegate Community Health Centre, Toronto, Canada; Suite 6042, Bond Wing, 30 Bond St., M5B 1W8 Toronto, ON Canada

**Keywords:** Asthma, Quality improvement, Spirometry, Before-and-after study design, Mentorship

## Abstract

**Background:**

Asthma is among the most common chronic diseases in adults. International guidelines have emphasized the importance of regular spirometry for asthma control evaluation. However, spirometry use in primary care remains low across jurisdictions. We sought to design and evaluate a knowledge translation intervention to address both the poor quality of spirometry and the underuse of spirometry in primary care.

**Methods:**

We designed a 1-year intervention consisting of initial interactive education and hands-on training followed by unstructured peer expert mentoring (through an online portal, email, telephone, videoconference, fax, and/or in-person). We recruited physician and allied health mentees from across primary care sites in Ontario, Canada. We compared spirometry-related knowledge immediately before and after the 1-year intervention period and the quality of spirometry testing and the usage of spirometry in patients with asthma in the year before and the year of the intervention.

**Results:**

Seven of 10 (70 %) invited sites participated, including 25/90 (28 %) invited allied health mentees and 23/68 (34 %) invited physician mentees. We recruited 7 physician mentors and 4 allied health mentors to form 3 mentor-mentee pods. Spirometry knowledge scores increased from 21.4 +/− 3.1 pre- to 27.3 +/− 3.5 (out of 35) (*p* < 0.01) post-intervention. Spirometry acceptability and repeatability criteria were met by 59/191 (30.9 %) spirometries and 86/193 (44.6 %) spirometries [odds ratio 1.7 (1.0, 3.0)], in the pre-intervention and intervention periods, respectively. Spirometry was ordered in 75/512 (14.6 %) and 129/336 (38.4 %) respiratory visits (*p* < 0.01), and in 20/3490 (0.6 %) and 36/2649 (1.4 %) non-respiratory visits (*p* < 0.01), in the pre-intervention and intervention periods, respectively.

**Conclusions:**

A mentorship-based intervention involving physicians and allied health team members can enhance knowledge, quality, and actual use of spirometry in real world primary care settings. A future controlled study should assess the impact of this intervention on patient outcomes, its cost-effectiveness, and its sustainability.

**Electronic supplementary material:**

The online version of this article (doi:10.1186/s12890-016-0220-6) contains supplementary material, which is available to authorized users.

## Background

Asthma is among the most common chronic diseases in adults, affecting 7.9 % of the US population, increasing in prevalence [1], and carrying an annual economic burden of $18 billion [2]. International guidelines have emphasized the importance of spirometry for both asthma diagnosis and control evaluation at regular intervals [3–6].

Spirometry can be effectively and efficiently performed in outpatient primary care settings [7, 8] and office spirometry with portable, handheld spirometers may be preferable to pulmonary function lab referral, as it reduces time and travel burdens for patients and eliminates diagnostic delays for physicians [9, 10]. Regular use of spirometry has also been shown to decrease asthma overdiagnosis [9, 11, 12] and to improve adherence to guideline-based pharmacological and non-pharmacological therapies in primary care [8].

However, spirometry use remains low across jurisdictions [13]. In a Canadian survey, only 35 % of patients with asthma reported having ever had spirometry, and only 46 % of primary care physicians (PCPs) reported using spirometry to monitor asthma [14]. An administrative database analysis of 485 866 newly diagnosed asthmatics confirmed that only 42.6 % had spirometry around the time of diagnosis [13]. Reported reasons for this underuse among primary care physicians include limited access to spirometry, lack of spirometry interpretation skills, and concerns about the quality of in-office spirometry [15, 16]. Indeed, prior reports have identified poor coaching, poor effort, an inaccurate spirometer, and/or inappropriate interpretation as factors contributing to erroneous results and potentially harmful patient misclassifications [17, 18].

We sought to design and evaluate a knowledge translation intervention to address the underuse of spirometry in primary care in Canada. Herein, we describe this multi-faceted intervention and its effects on knowledge of spirometry performance and interpretation, quality of spirometry performance, and usage of spirometry in primary care.

## Methods

### Study design

This was a pre- and post-analysis, comparing outcomes during a 1-year intervention period to those in the year prior. As a pragmatic quality improvement study intending to measure real-world effectiveness, we sought to design an intervention and evaluation which would entail minimal changes to clinical workflow.

### Intervention (Fig. 1)

**Fig. 1:**
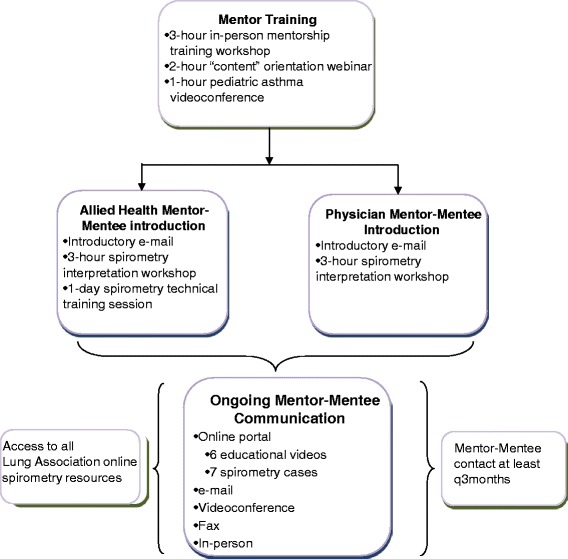
Intervention Schematic. Members of each mentor-mentee pod met in person at least once within the first 3 months of the start of the intervention, and the subsequent frequency and nature of mentor-mentee interactions was determined by participants themselves. However, mentors were asked to initiate contact with each mentee at least once every 3 months if no contact was ongoing. Brief educational videos (each featuring a mentor) and spirometry cases with corresponding questions were emailed to mentees and posted to the online portal every 4–6 weeks, to encourage mentor-mentee interaction. Mentors posted correct answers 1 week after each case was first posted. All users were also provided with access to the Ontario Lung Association’s (OLA) online spirometry-related educational resources throughout the intervention period (available at www.on.lung.ca)

In designing the intervention, we espoused the Knowledge-to-Action Framework [19], whereby we ascertained barriers and facilitators to primary care spirometry from end-users and used these to choose and tailor the intervention in conjunction with these end-users. Barriers and facilitators were ascertained through an electronic questionnaire, followed by an in-person workshop with primary care team members, during which we also collaboratively designed the intervention. Key identified barriers included limited knowledge of spirometry performance (among allied health staff performing spirometry) and interpretation techniques (among clinicians interpreting spirometry), and limited access to specialist opinions for complex spirometries and/or clinical questions. Accordingly, we conceived of an intervention which linked peer experts [physicians with asthma expertise and certified respiratory educators (CREs) with spirometry expertise] with their primary care counterparts, in a mentorship model.

We conceived of mentor-mentee “pods,” each including physician and allied health mentors and corresponding mentees. Mentor types in each pod included: 1) an adult respirologist; 2) a pediatric respirologist; 3) a PCP with asthma expertise; and 4) a CRE with spirometry expertise. Mentors and mentees in each pod were invited to communicate through the following information and communications technology (ICT) tools: an online portal - a web-portal enabling resource sharing and secure communication (users could also see the content of previous conversations for reference; responses to posted questions were guaranteed within a 24-h turnaround; and registered users received updates on any new portal content via an RSS feed and an automated e-mail); email - mentees could e-mail any questions to their mentors and were guaranteed a 24-h turnaround; videoconference - mentors and mentees had access to videoconferencing equipment; and the following classical communications tools: telephone - mentees could make personal calls to a mentor, and/or could call a dedicated hotline to leave a voice message for a mentor; teleconference - mentors and mentees had access to a free teleconference line to set up group teleconferences; fax - mentees could fax mentors spirometries and/or specific questions for assistance; and in-person meetings - mentees and mentors were encouraged to plan in-person meetings as convenient. Mentor-mentee interaction points are detailed in Fig. 1.

### Population and recruitment

We identified primary care sites that were part of the Ontario Primary Care Asthma Program (PCAP) (a provincially funded asthma quality improvement program), and sampled these purposively to represent a wide range of service models and environments (rural, urban sites, and sites serving underserviced populations). Included sites required at least one on-site certified asthma or respiratory educator, on-site spirometry capability, access to videoconferencing, and current use of an electronic medical record (EMR) system. Sites were excluded if they were involved in any other asthma quality improvement program.

We identified mentors among current and previous teachers involved in Ontario Lung Association (OLA) asthma-related educational activities. Physician mentors had to be recognized respiratory disease opinion leaders within the local community, and allied health mentors had to possess a strong knowledge base around spirometry technical training. Based on our prior experience [20], we pre-determined a mentor-mentee ratio of no larger than 1 to 5. Mentors were reimbursed for time spent on mentorship activities, according to the Ontario College of Family Physicians remuneration agreement for physician mentors ($150/h) and OLA remuneration policies for allied health mentors ($50/h). All mentors completed an in-person mentorship training workshop and participated in workshops focused on spirometry-related content before intervention launch (Fig. 1).

### Outcomes

#### Spirometry knowledge

Given a lack of validated tools for measuring spirometry knowledge, we designed a knowledge test (self-administered anonymously online or in-person within 3 months before intervention launch and 3 months after the intervention period). This included 5 sample spirometries testing interpretation (interpretation knowledge component), including a patient with asthma, COPD, lung restriction, a normal study, and an inadequately performed study. It also included multiple-choice and short-answer questions testing knowledge of spirometry performance quality standards (performance knowledge component). This test (Additional file 1) was based on the PCAP spirometry interpretation program, accredited by the College of Family Physicians of Canada.

#### Quality of spirometry testing

We collected a random sample of 2 spirometries per month performed by allied health mentees who routinely performed spirometry, in the year prior to and the year of the intervention. This sample size was based on estimates of baseline spirometry volumes at each site and on the feasibility of detailed spirometry analysis. A registered cardiopulmonary technologist with experience in spirometry evaluation [21] was masked to the intervention period, and compared each spirometry for compliance with American Thoracic Society/European Respiratory Society standards for spirometry performance and reporting (please see legend, Table 1 for details) [22].

**Table 1:** Quality of Spirometry Performance and Reporting

Criterion	Pre-Intervention	During Intervention	Mean difference	Odds Ratio	*p*-value
Total spirometries	210	208	-	-	-
Mean No. of blows per spirometry	3.8 (3.5, 4.1)	3.7 (3.4, 4.0)	−0.08 (−0.25, 0.09)	-	0.37
Mean No. of acceptable^a^ blows per spirometry	1.9 (1.3, 2.5)	2.1 (1.5, 2.7)	0.22 (−0.07, 0.50)	-	0.16
Probability of spirometries with ≥3 acceptable blows	0.36 (0.16, 0.63)	0.49 (0.27, 0.72)	-	1.7 (1.1, 2.6)	0.03
Probability of spirometries with ≥2 repeatable blows^b^	0.85 (0.74, 0.92)	0.93 (0.85, 0.97)	-	2.2 (1.0, 4.6)	0.05
Probability of blows with a poor start	0.06	0.05	-	0.74 (0.35, 1.6)	0.58
Probability of blows with an unsatisfactory exhalation	0.39	0.35	-	0.84 (0.62, 1.1)	0.30
Probability of blows with significant artifact	0.13	0.14	-	1.2 (0.86, 1.6)	0.34
Probability of reporting correct FVC^c^	0.71 (0.57, 0.85)	0.78 (0.66, 0.90)	-	1.43 (0.72, 2.9)	0.35
Probability of reporting correct FEV1^c^	0.73 (0.61, 0.85)	0.79 (0.68, 0.89)	-	1.4 (0.62, 3.0)	0.45
Probability of documenting inadequate spirometry^d^	0.08 (0, 0.18)	0.12 (0, 0.18)	-	1.4 (0.73, 2.8)	0.46
Probability of identifying appropriate reason for inadequate spirometry^e, f^	0.02 (0, 0.07)	0.06 (0, 0.13)	-	2.4 (0.67, 8.8)	0.20
Probability that spirometer was calibrated before test	0.96 (0.68, 1.0)	0.97 (0.77, 1.0)	-	1.4 (1.1, 1.9)	0.01
Probability that spirometry met both acceptability and repeatability criteria	0.32 (0.14, 0.58)	0.45 (0.25, 0.67)	-	1.7 (1.0, 3.0)	<0.05

95 % confidence intervals are provided in brackets

CI denotes confidence interval

^a^“acceptable” was defined by the absence of a poor start [i.e. an extrapolated volume < 5 % of FVC or 150 mL (whichever was greater)], a satisfactory exhalation [defined by reaching a plateau in the volume–time curve, with no change in volume (<0.025 L) for ≥ 1 s], and absence of any significant artifact (including evidence of cough during the first second of exhalation, glottis closure that influences the measurement, early termination or cut-off, submaximal effort, leak, or an obstructed mouthpiece) [22]

^b^among spirometries with at least 2 acceptable blows (a spirometry was considered “repeatable” when the two largest FVC values and the two largest FEV1 values were each within 150 mL of each other) [22]

^c^among spirometries with at least 1 acceptable blow

^d^Technician comments were searched for documentation that the test was inadequate (among spirometries which did not meet ATS criteria of at least 3 acceptable and 2 repeatable blows) [22]

^e^Technician comments were searched for documentation of the reason that the test was inadequate (among spirometries which did not meet ATS criteria of at least 3 acceptable and 2 repeatable blows) [22]

^f^For this outcome, applying the bootstrap method to estimate standard errors failed to converge in one-third of the replications. Therefore, results are reported using robust standard errors

#### Spirometry usage

Spirometry usage was ascertained through a retrospective electronic chart review across the 4 project sites with compatible EMR systems (this analysis was not possible in the other 3 sites). We included patients ≥6 years old who had been seen by a physician or nurse practitioner mentee in the time period of interest, excluded patients with COPD, and characterized the reason for each visit as respiratory or non-respiratory.

### Analysis

Baseline knowledge questionnaire scores were compared between user types using a ttest, and individuals’ pre/post scores were linked through a unique identifier and compared with a paired ttest. The mean number of blows per spirometry and mean number of acceptable blows per spirometry were estimated using Generalized Estimating Equations with normal distribution and identity link, accounting for the repeated measures structure of the study, assuming exchangeable correlation. The proportion of spirometries with ≥ 3 acceptable blows; with ≥ 2 reproducible blows; for which the spirometer was calibrated; and which met both acceptability and repeatability criteria were calculated using the GLIMMIX procedure, which enables Generalized Estimating Equations with small-sample corrections. For other spirometry quality criteria, Generalized Estimating Equations with the binomial distribution and logit link were used to estimate the odds ratios, predicted probabilities, and 95 % confidence intervals, accounting for the repeated measures design, assuming exchangeable correlation [using Stata 13 (StataCorp LP, Texas, USA)]. We compared the proportion of visits on which spirometry was ordered between pre-intervention and intervention periods using a chi square test or Fisher’s Exact test (as appropriate). P-values <0.05 were considered significant, and analyses were performed with SAS 9.4 (SAS Institute Inc., NC, USA) unless stated otherwise.

## Results

### Recruitment

**Fig. 2:**
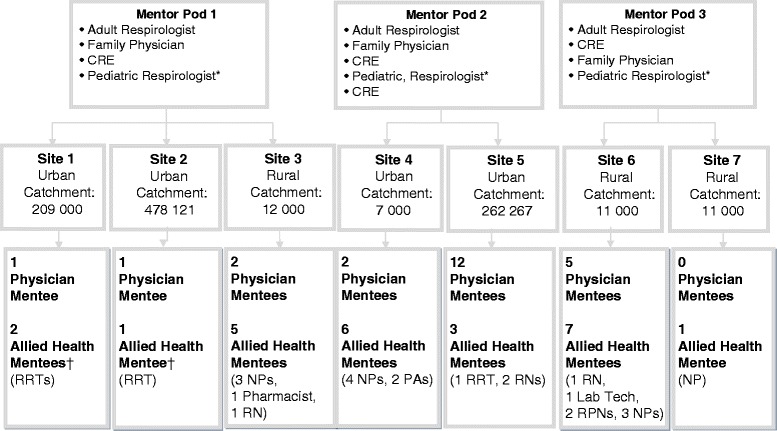
Primary Care Sites and Mentor-Mentee Pods. * the pediatric respirologist was shared between the 3 pods. † One Registered Respiratory Therapist was shared between sites 1 and 2 (performed spirometry at both sites). RRT denotes Registered Respiratory Therapist; NP denotes Nurse Practitioner; PA denotes physician assistant; RN denotes Registered Nurse; RPN denotes Registered Practical Nurse

Seven of 10 (70 %) invited PCAP sites agreed to participate (Fig. 2). Among the three sites which chose not to participate, one expressed an initial interest but declined due to insufficient human resources for data extraction from the electronic medical record system, another cited fatigue with quality improvement initiatives, and another did not provide a reason. Across recruited sites, 25/90 (28 %) allied health team members and 23/68 (34 %) physicians consented for the program (48 mentees total; Fig. 2). Mentees had been in practice for 17.2 +/− 13.0 years (range 1 month - 39 years), and 57 % were female. Among 40 mentees reporting on previous spirometry training experience, 24 (60 %) had none, 9 (22 %) had participated in at least one in-person spirometry course, 5 (12 %) had attended a hands-on spirometry technique workshop, and 2 (5 %) had completed an online course only.

We recruited 7 physician mentors and 4 allied health mentors to form 3 pods (Fig. 2). Mentors had been in practice for 26.6 +/− 8.0 years (range 13–40 years).

### Outcomes

#### Spirometry knowledge

Twenty-five participants completed the pre-intervention questionnaire. The mean baseline score for spirometry interpretation knowledge was 12.0 +/− 5.3 (out of 21), and for spirometry performance knowledge was 7.9 +/− 2.3 (out of 14), giving a total score of 19.9 +/− 5.5 (out of 35). Interpretation knowledge scores tended to be higher among those participants who interpreted spirometry compared to those who did not (12.9 +/− 1.0 vs 11.6 +/− 6.4, *p* = 0.45), and performance knowledge scores were higher among those who performed spirometry compared to those who did not (9.9 +/− 2.2 vs 6.9 +/− 1.8, *p* < 0.01) (supporting the questionnaire’s construct validity). All participants who completed both pre- and post-intervention questionnaires showed improvement, with a mean increase in score from 21.4 +/− 3.1 to 27.3 +/− 3.5 (*p* < 0.01). However, it should be noted that use of anonymous questionnaires prevented us from adjusting this analysis for any possible effects of nesting within study sites and/or mentor pods, and therefore p-values may be liberal.

#### Quality of spirometry testing

We analyzed 418 tests (1438 blows) performed by 9 allied health mentees (mean of 23.3+/− 3.6 spirometries pre-intervention and 23.1+/− 5.1 spirometries during intervention, per operator). Both acceptability and repeatability criteria were met by 59/191 (30.9 %) spirometries and 86/193 (44.6 %) spirometries [odds ratio 1.7 (1.0, 3.0)], in the pre-intervention and intervention periods, respectively (Table 1).

#### Spirometry usage

Spirometry was ordered in 75/512 (14.6 %) and 129/336 (38.4 %) of respiratory visits (*p* < 0.01), and in 20/3490 (0.6 %) and 36/2649 (1.4 %) of non-respiratory visits (*p* < 0.01), in the pre-intervention and intervention periods, respectively. Individual site data are shown in Table 2.

**Table 2:** Pre-Intervention and During Intervention Spirometry Usage in Patients with Asthma

Site	1		2		3		5	
	Pre	During	Pre	During	Pre	During	Pre	During
Total patients (under the care of mentees) (≥6 years of age)	1499	1594	2814	2838	5686	5647	10806	10806
Patients with asthma	83^i^	90^i^	269^i^	322^i^	264^j^	224^j^	1655^k^	1677^k^
Proportion of visits for respiratory complaints^l^ in which spirometry was ordered (%)	6/40 (15 %)	29/32 (91 %)^a^	19/131 (14 %)	15**/**55 (27 %)^b^	35/141 (25 %)	26/113 (23 %)^c^	15/200 (7.5 %)	59/136 (43 %)^d^
Proportion of visits for non-respiratory complaints in which spirometry was ordered (%)	4/156 (2.6 %)	21/269 (7.8 %)^e^	4/706 (0.6 %)	0/601 (0 %)^f^	5/896 (0.6 %)	9/602 (1.5 %)^g^	7/1732 (0.4 %)	6/1177 (0.5 %)^h^

Please see Fig. 2 for individual site charactersitics

^a^ *p* < 0.01; ^b^ *p* = 0.04; ^c^ *p* = 0.74; ^d^ *p* < 0.01; ^e^ *p* = 0.03; ^f^ *p* = 0.13; ^g^ *p* = 0.06; ^h^ *p* = 0.68 *please note that these values were not corrected for any possible effects of nesting within mentor pods

^i^identified through a free-word “asthma” used in the clinical chart; or “asthma” in the problem list or past health history; or use of the asthma billing code in last 1 year [37], followed by a manual review to remove all patients who did not have a clinical diagnosis of asthma and/or who had a clinical diagnosis of COPD

^j^Identified through a free-word “asthma” used in the clinical chart; or “asthma” in the problem list or past health history [37], followed by a manual review to remove all patients who did not have a clinical diagnosis of asthma and/or who had a clinical diagnosis of COPD

^k^identified through a search for patient with an active prescription for an asthma medication, followed by removal of any patients in whom a COPD billing code had been used in the past or who had “COPD” in the problem list or past health history [37]

^l^visits where the chief complaint was cough, wheeze, short of breath, or upper/lower respiratory tract infection

## Discussion

We designed a knowledge translation intervention which included interactive education, hands-on training and ongoing mentorship, and improved spirometry knowledge, the quality of spirometry, and the frequency of its use for patients with asthma in primary care.

### Spirometry knowledge

As in prior studies, we identified a lack of knowledge of spirometry performance and interpretation as major barriers to its uptake in primary care [15, 16]. Accordingly, improvements in knowledge are a likely pre-requisite for increased spirometry usage. In a prior study, Eaton, et al. demonstrated similar improvements in knowledge among both physicians and practice nurses immediately after a spirometry workshop. However, some of these knowledge gains were lost when subjects were re-tested 12 weeks later [23]. In our study, in-person educational sessions occurred in the first 3 months of the intervention, yet sustained knowledge gains were observed after one year. This may suggest that the ongoing mentorship component enabled reinforcement of and/or further knowledge gain over time. Sample sizes were too small to ascertain differences in knowledge acquisition (and type of knowledge acquired) between physician and allied health mentees, and a larger future study should address this.

### Quality of spirometry testing

Concern among ordering physicians about the quality of spirometry is another important barrier to its usage [15, 17, 18, 24]. Our intervention demonstrated quality improvements which were comparable to those in previous reports. A small United Kingdom study of audit and feedback followed by a didactic lecture by a specialist and a hands-on session with a specialized nurse dramatically reduced spirometry technical errors among primary care GPs and nurses [25]. In New Zealand, Eaton, et al. demonstrated significant improvements in spirometry technical competence among primary care physicians and nurses in the 12 weeks after a 2-h spirometry workshop [23]. In contrast, a 70-min CD-ROM-based tutorial alone was shown to be ineffective [26], whereas remote delivery through telemedicine [27, 28] or web-based interactive meetings [29] were effective. Our findings are congruent with this prior literature, and support the need for personalized training and ongoing feedback as part of an effective intervention [30].

However, it should be noted that there is no consistent threshold technical accuracy rate for spirometry in primary care, and previously reported rates vary widely [8, 16]. Eaton, et al. [23] reported baseline proportions with ≥ 3 acceptable blows (5.1 %) and ≥ 2 repeatable blows (3.4 %) which were much lower than those in our study, and improved to only 18.9 % and 13.5 %, respectively [23]. In contrast, a large Spanish public health telemedicine-based training and quality assurance program achieved 84 % spirometry acceptability rates at 9 months in a cost- effective fashion [31], though baseline acceptability rates were also much higher than ours (61 %) [28]. The Finnish Asthma Program achieved 80 % technical adequacy through extensive training and a suite of implementation initiatives [7], and a Canadian program demonstrated acceptability and repeatability in 71 % of tests after a program featuring workshops, supervised patient sessions, and ongoing access to trainers [32]. It is possible that the smaller improvements seen in our study were related to both the smaller volumes of spirometry in several of our centers (impeding volume-based technical skills acquisition), and the fact that nurses and asthma educators were uniformly responsible for spirometry in the described Finnish and Canadian programs, respectively, whereas in our study, spirometries were performed by personnel with varied clinical duties and training (Fig. 2), which may have affected their ability to sustain a high level of technical competence. The quality and usability of the spirometer itself (including provision of quality warnings and guidance) may also influence technical competence [16, 30].

It should also be noted that although we demonstrated significant improvements in test acceptability and repeatability with our intervention, several other individual quality metrics showed trends towards improvements without reaching statistical significance (Table 1). Several factors may account for this. The acceptability of a blow is a function of a good start, a satisfactory exhalation, and a lack of artifact; sample sizes may have been too small to show improvements in these individual components. These patient-dependent variables may also each have been less likely to change with our professional-directed intervention. Furthermore, acceptability and repeatability were given major emphasis in educational and training sessions, as they are the fundamental determinants of a test’s validity, and hence its interpretability [22]. Correspondingly, other aspects of overall spirometry quality such as reporting and documentation may have been less likely to improve.

### Spirometry usage

We demonstrated a significant increase in spirometry usage during the intervention period. Although novel models to deliver spirometry, such as nurse-led community respiratory assessment units have been studied [33], previous studies have not demonstrated comparable, sustained increases in the use of onsite in-office spirometry in real world primary care settings. In an Australian cluster randomized controlled trial, six hours of combined theory and practice-based spirometry training for PCPs and nurses did not increase the use of spirometry, nor impact patient outcomes [34]. This suggests that addressing knowledge and competence may be insufficient, and a more complex and sustained intervention such as ours is likely required. In a previous study, the availability of spirometry data altered primary care asthma management in nearly half of patients (including medication changes) [8], suggesting that this has an important impact on care. A future study should document changes in clinical management and patient outcomes to prove the clinical impact of our intervention.

Despite significant improvements with the intervention, overall spirometry usage remained below 40 %, even during visits for respiratory complaints. This is likely because the barriers and facilitators to spirometry use are multi-faceted and complex, and correspondingly different between sites. This is supported by the observed variability in baseline rates and effect sizes between sites, with spirometry ordering during visits for respiratory complaints ranging from 23 % to 91 % (by site) in the intervention period (Table 2). Several barriers other than those addressed by our intervention (interpretation knowledge and poor test quality) may play a role. For example, system-level barriers related to workflow and availability of personnel for spirometry may be factors which vary between sites. Also, some primary care physicians may not believe that spirometry is required for asthma diagnosis and follow-up, as has been observed in qualitative studies of barriers to spirometry usage in COPD [35, 36]. Future studies should correlate spirometry usage in each site with baseline site-specific barriers and facilitators and site-specific effects of the intervention on these factors.

The main strength of our study was the engagement of end-users and consideration of self-reported barriers in intervention design [19]. We identified a lack of knowledge of spirometry performance and interpretation, and poor spirometry quality as major barriers to uptake in primary care. Accordingly, measured improvements in these parameters provide mechanistic support for the demonstrated increase in spirometry uptake. We also present a novel intervention which leveraged modern communication modalities, included both physicians and allied health team members, and can be replicated in diverse settings.

Our study has several limitations. Our pre/post design is susceptible to confounding by other causes. Although we are not aware of any other initiatives which would be expected to change clinician behaviour during the study period, our results will require validation in a controlled trial. We recruited sites of various sizes and settings, however our findings can not be generalized to settings without allied health personnel who can be trained to perform spirometry. A minority of health care professionals that were approached agreed to participate in the program. Recruitment was conducted by a local site leader, and may have been more successful if potential mentees were approached directly by mentors (opinion leaders), ideally through a web-based recruitment tool explaining potential program benefits. Such a recruitment tool could be employed in a future rollout of this intervention, and could highlight the positive outcomes seen in this study. The robust measurement built around this project also required numerous and extensive questionnaires, which likely further deterred participation, and would not be an issue in a future rollout. Finally, we did not perform a concurrent cost analysis and cannot comment on the cost-benefit ratio of the intervention. However, a prior study demonstrated that improvements in the quality of spirometry performance were associated with a 50 % reduction in referrals to a respirologist [25]. This suggests that increased usage of and provider confidence in spirometry may reduce the need for referrals. Given the significant cost of full referrals when compared to the telephone/internet consultations enabled by our intervention, this may present significant cost savings for health care systems as well as time and travel cost savings for patients. Costs should be evaluated in a future study.

## Conclusions

In summary, a mentorship-based intervention involving both primary care physicians and allied health team members can enhance knowledge, quality, and use of spirometry in eclectic, real world primary care settings. Measuring the effects of such a program on patient outcomes, its costs, and the sustainability of its effects will be important considerations in a future controlled study.

### Ethics and consent to participate

The study was approved by the St. Michael’s Hospital Institutional Review Board. Written informed consent was obtained from each subject.

### Availability of data and materials

Authors would be pleased to consider requests to share original study data.

## Additional file

Additional file 1: Spirometry Knowledge Test. Spirometry knowledge test used in the study. (DOC 765 kb)

## Abbreviations

CRE: certified respiratory educator
EMR: electronic medical record
OLA: ontario lung association
PCAP: primary care asthma program
PCP: primary care physician
