# Supplementary material for: Effects of a 12-month multi-faceted mentoring intervention on knowledge, quality, and usage of spirometry in primary care: a before-and-after study
Source: BMC Pulm Med. 2016 Apr 21;16:56. doi: 10.1186/s12890-016-0220-6 (PMC4839111; doi:10.1186/s12890-016-0220-6)
Supplement: Additional file 1: — Spirometry Knowledge Test. Spirometry knowledge test used in the study. (DOC 765 kb) [file 12890_2016_220_MOESM1_ESM.doc]

**Background Information**

1. **What is your professional designation?**

| Physician |  | Registered Respiratory Therapist |  | Pharmacist |  |
| --- | --- | --- | --- | --- | --- |
| Nurse Practitioner |  | Pulmonary Function Technologist |  | CRE |  |
| Registered Nurse |  | Executive Director |  | CAE |  |
| Other (please specify): | |  |  |  | |

1. **Please select the type of setting. (Check all that apply)**

|  | FHT |  | CHC |  | Hospital |
| --- | --- | --- | --- | --- | --- |
|  | Rural |  | Urban |  | First Nations |
|  | Other (please specify): | |  |  |  |

1. a) Please indicate whether you have participated in the following spirometry programs?

| **Spirometry Programs** | **Program Taken** | **When did you participate in the program? Please check** | | | | |
| --- | --- | --- | --- | --- | --- | --- |
|  |  | 0-6 months | 6-12 months | 1 year | 2 years | 3+ years |
| PCAP Spirometry Orientation | ** Yes  No** |  |  |  |  |  |
| PEP Spirometry Interpretation Program | ** Yes  No** |  |  |  |  |  |
| Spirometry in Primary Care CD-Rom/ Online Version | ** Yes  No** |  |  |  |  |  |
| SpiroTrec Training | ** Yes  No** |  |  |  |  |  |

**b) If you have participated in any other spirometry program, please indicate which program, and when you participated:** **__________________________________________________________________________________________________________________________________________________________________________________________________________________________________________**

1. **a) Does your site have access to spirometry testing?**

**** **Yes  No**

1. **If yes then how does your site utilize spirometry?**

** Perform spirometry  Refer patients for spirometry**

1. **Do you perform spirometry yourself?**

**** **Yes  No**

1. **Do you interpret spirometry yourself?  Yes  No**

**CASE STUDIES**

# Table 1: Degrees of Obstruction (Modified ATS/ERS criteria)

| **Severity of Obstruction:** |  |
| --- | --- |
| **FEV_1_** |  |
| Mild | >70% predicted |
| Moderate | 50% to 69% predicted |
| Severe | <50% predicted |
| **Severity of Restriction:** |  |
| **FVC** |  |
| Mild | 65% to 80% predicted |
| Moderate | 50% to 65% predicted |
| Severe | <50% predicted |

**Case 1**

Ronald is a 42-years-old caucasian, male, teacher with no history of smoking. For the past 5 years he has been experiencing shortness of breath associated with exercise.


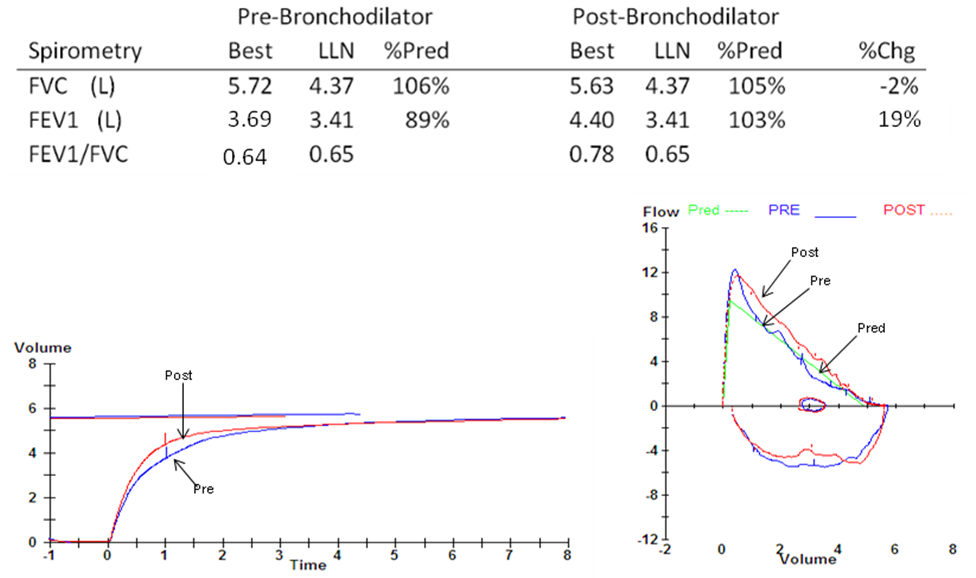


1. **Is this an acceptable test?**

** Yes  No**

1. **If no, please explain why not.**

________________________________________________________________________

_________________________________________________________________________

**3. If yes, please interpret the spirometry results (refer to Table 1).**

**Comment on:**

1. **Presence of obstruction or restriction** _____________________________________________________________________________

_____________________________________________________________________________

1. **Severity of any obstruction or restriction**

_____________________________________________________________________________

_____________________________________________________________________________

1. **Response to bronchodilator (significant versus non-significant)**

_____________________________________________________________________________

_____________________________________________________________________________

1. **Likely diagnosis ______________________________________________________________**

**Case 2**

Claire is a 55-year-old smoker (36 pack-years) who works as a legal assistant and has suffered from intermittent cough, wheezing and breathlessness on exertion for 2-3 years.


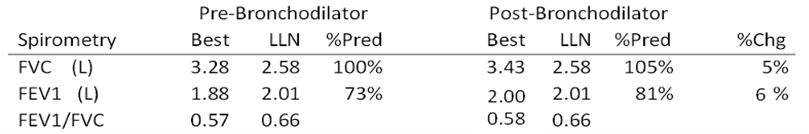


**4. Is this an acceptable test?**

** Yes  No**

**5. If no, please explain why not.**

________________________________________________________________________

_________________________________________________________________________

**6. If yes, please interpret the spirometry results (refer to Table 1).**

**Comment on:**

1. **Presence of obstruction or restriction** _____________________________________________________________________________

_____________________________________________________________________________

1. **Severity of any obstruction or restriction**

_____________________________________________________________________________

_____________________________________________________________________________

1. **Response to bronchodilator (significant versus non-significant)**

_____________________________________________________________________________

_____________________________________________________________________________

1. **Likely diagnosis ______________________________________________________________**

**Case 3**

Aaron is an 18 year old male student who coughs after playing hockey 2-3 times a week.

Date: 2007 03 16 Subject: BC Gender: Male Age: 18

Height (cm): 169 Weight (kg): 70.0 Race: Asian


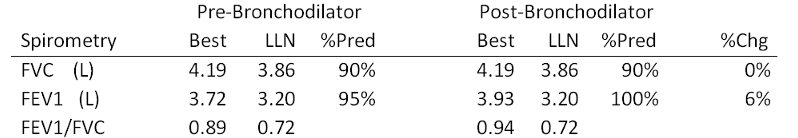


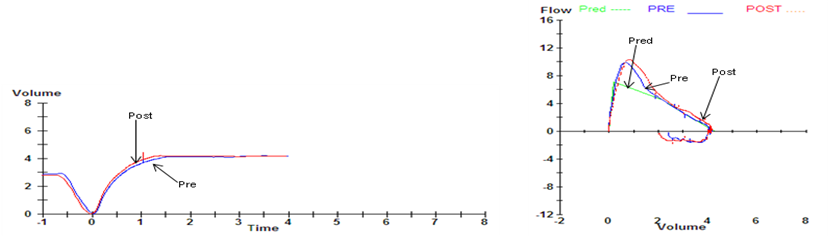


**7. Is this an acceptable test?**

** Yes  No**

**8. If no, please explain why not.**

________________________________________________________________________

_________________________________________________________________________

**9. If yes, please interpret the spirometry results (refer to Table 1).**

**Comment on:**

1. **Presence of obstruction or restriction** _____________________________________________________________________________

_____________________________________________________________________________

1. **Severity of any obstruction or restriction**

_____________________________________________________________________________

_____________________________________________________________________________

1. **Response to bronchodilator (significant versus non-significant)**

_____________________________________________________________________________

_____________________________________________________________________________

1. **Likely diagnosis ______________________________________________________________**

**Case 4**

Wan is a 34 year old male working in the granite fabrication and installation business for over 10 years. He complains of a chronic cough, fatigue and weakness.

Date: 2004 07 14 Subject: RP Gender: Male Age: 34

Height (cm): 182 Weight (kg): 77.3 Race: Caucasian


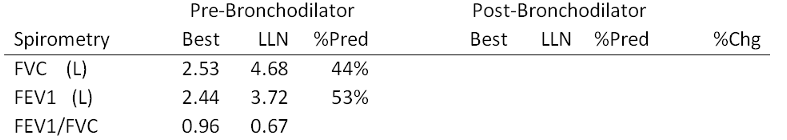


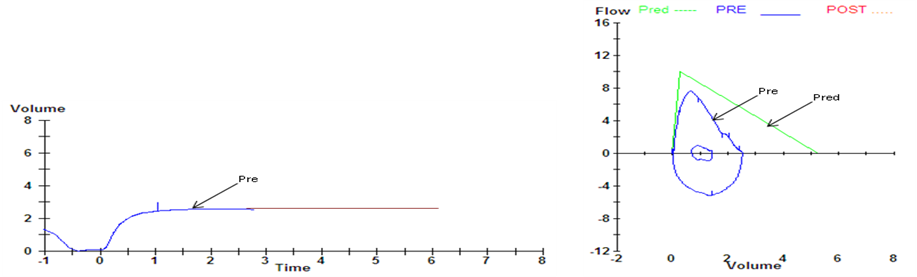


**10. Is this an acceptable test?**

** Yes  No**

**11. If no, please explain why not.**

________________________________________________________________________

_________________________________________________________________________

**12. If yes, please interpret the spirometry results (refer to Table 1).**

**Comment on:**

1. **Presence of obstruction or restriction** _____________________________________________________________________________

_____________________________________________________________________________

1. **Severity of any obstruction or restriction**

_____________________________________________________________________________

_____________________________________________________________________________

**Case 5**

Susan is a 43 year old early childhood educator, who has seasonal allergies and frequent colds.


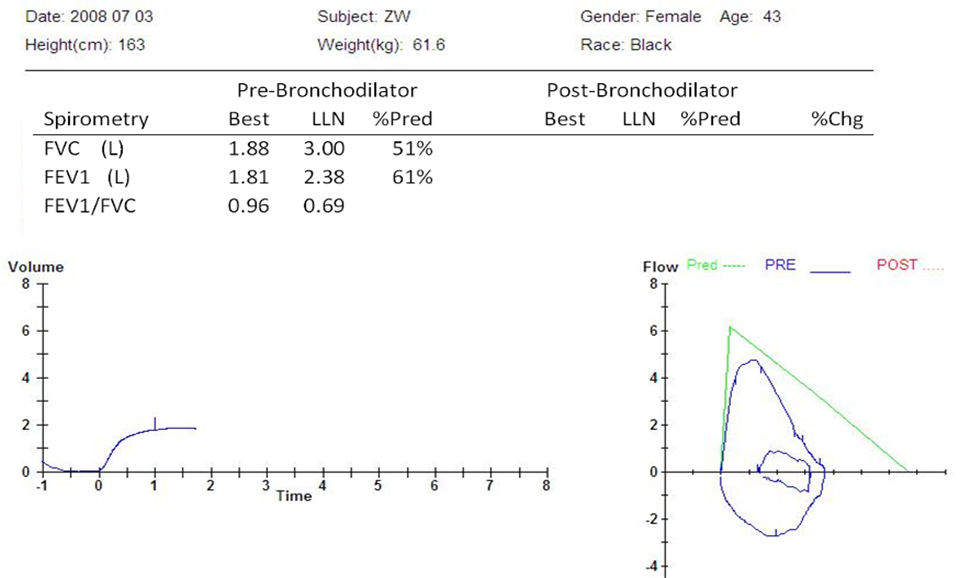


**13. Is this an acceptable test?**

** Yes  No**

**14. If no, please explain why not.**

________________________________________________________________________

_________________________________________________________________________

**15. If yes, please interpret the spirometry results (refer to Table 1).**

**Comment on:**

1. **Presence of obstruction or restriction** _____________________________________________________________________________

_____________________________________________________________________________

1. **Severity of any obstruction or restriction**

_____________________________________________________________________________

_____________________________________________________________________________

**SPIROMETRY PERFORMANCE AND INTERPRETATION**

1. **What are the criteria for bronchodilator reversibility?**

At least ____% and _____cc post-bronchodilator improvement in FEV1.

1. **Circle all options listed below that are contraindications to performance of spirometry:**
2. morbid obesity
3. hemoptysis of unknown origin
4. thoracic, abdominal, or cerebral aneurysms
5. cough productive of green sputum
6. recent eye, thorax, or abdomen surgery
7. **The following is used to determine patient predicted values. (Check all that apply)**

- Age
- Height
- Weight
- Race
- Gender

1. **The minimum duration of a forced expiratory maneuver for a spirometry to be valid is** ____ **seconds.**
2. **How often should a spirometer be calibrated for volume?** _____________________________
3. **Choose the statement that describes the best position for spirometry:**
4. Standing with legs straight
5. Standing with knees slightly bent
6. Sitting with feet slightly off the ground
7. Sitting with feet on the ground
8. Sitting with legs crossed
9. **What is the minimum number of acceptable maneuvers required for a spirometry test to be considered reliable?** ____________________________________________________________
10. **For spirometry results to be considered reproducible,**
11. the two largest FVC’s should not vary by more than _____ ml
12. the two largest FEV1’s should not vary by more than _____ ml
13. **When reporting the FEV1 and the FVC, the following should be used:**
14. The FEV1 and FVC from the acceptable maneuver with the largest FEV1
15. The FEV1 and FVC from the acceptable maneuver with the largest FVC
16. The FEV1 and FVC from the acceptable maneuver with the highest FEV1/FVC ratio
17. The largest FVC and the largest FEV1 from any acceptable maneuvers
18. The means of the FEV1s and FVCs from all acceptable maneuvers

**Consent:**

- I allow the information contained in my Pre-Assessment to be used for evaluation research. I understand that all data collected will be kept confidential.
- I do not wish my information to be used for the purpose of evaluation research.
